# Effects of supplementary feeding on the rumen morphology and bacterial diversity in lambs

**DOI:** 10.7717/peerj.9353

**Published:** 2020-06-19

**Authors:** Feng Lv, Xiaojuan Wang, Xin Pang, Guohua Liu

**Affiliations:** College of Animal Science and Technology, Gansu Agricultural University, Lanzhou, China

**Keywords:** Hu lambs, Rumen, Microbiota, Milk replacer, Starter, High-throughput sequencing, Rumen liquild, Supplementary feeding, Bacterial diversity, Rumen morphology

## Abstract

Early supplementary feeding of lambs before weaning is important to meet their nutritional needs, promote the development of rumen and improve performance. To study the effect of early supplementary feeding on rumen development and the microbiota of lambs, 22 Hu lambs were randomly divided into two groups: one group was fed with milk replacer (group C), and the other group was fed with milk replacer and starter (group S). At 28 days, six lambs in each group were slaughtered, and the rumen content and tissue samples were collected for detection and analysis. The starter significantly promoted the length of rumen papilla (*P* = 0.03), the concentration of acetate, propionate, butyrate and total volatile fatty acids (TVFA) (*P* < 0.01), which were higher in group S compared with group C. Group C had a higher rumen microbial diversity than group S. The dominant bacteria in the two groups were the same (Bacteroidetes, Firmicutes and Proteobacteria); however, they differed notably at the genus level. The microbial abundance of the two groups was significantly different for 22 species. In group C, the first three dominant bacteria were *Bacteroides, Porphyromonas*, and *Campylobacter*, while in group S they were *Succinivibrio, unidentified_Prevotellaceae*, and *unidentified_Lachnospiraceae*. Spearman correlation analysis showed that some ruminal bacteria were closely related to internal environmental factors, e.g., the relative abundances of *unidentified_Bacteria*, *Euryarchaeota, Fusobacteria*, and *Gracilibacteria* correlated negatively with acetate, propionate, butyrate, and TVFA (*P* < 0.05), while the relative abundances of *Firmicutes* correlated positively with acetate, propionate, butyrate and TVFA (*P* < 0.05). *Bacteroidetes* correlated negatively with propionate, butyrate, and TVFA (*P* < 0.05); *Synergistetes* correlated negatively with acetate, propionate, and butyrate (*P* < 0.05); *Deinococcus-Thermus* correlated negatively with propionate, butyrate, and TVFA (*P* < 0.05); *Spirochaetes* correlated negatively with propionate and TVFA (*P* < 0.05); and *Elusimicrobia* correlated negatively with propionate and butyrate (*P* < 0.05). *Actinobacteria* and *Verrucomicrobia* correlated positively correlated with NH_3_-N. In conclusion, supplementary feeding of lambs before weaning promoted the development of rumen tissue morphology and rumen microorganisms.

## Introduction

Rumen is an important digestive organ of ruminants and plays an important role (Roh et al., 2007). Complex physiological changes occur in the rumen before it can fully rely on fiber-rich food (Heinrichs, 2005). Studies have shown that rumen development is affected by a variety of factors (Khan, Weary & Keyserlingk, 2011; Górka et al., 2011; Yang et al., 2015; Yang et al., 2018). Among the numerous factors, age and diet have been studied a lot, and the current research is not only reflected in the epigenetic properties, but also reflected in a much deeper molecular level, microbial and gene expression have become a hot topic. O’Hara et al. (2020) used the DNA amplification sequence of the 16S rRNA gene to evaluate the bacteria and archaebacteria dynamics in the rumen of cattle, and the results showed that the microbial composition of early rumen was influenced by the age of calves, diet and local environment. Yáñez Ruiz, Abecia & Newbold (2015) and Abecia et al. (2017) showed that different early life nutrition interventions could be used to change the pattern of microbial colonization in young animals. Belanche et al. (2019) also showed that nutrition interventions in early life determined the initial state of the rumen microbial community, but the persistence of these effects in later life was weak, with age and inherent dietary changes being the mainly driving factors. The rumen microbiota is a complex and changeable system, and its intricate relationship needs to be explored for a long time.

With the development of sheep breeding industry, increasing in the reproduction rate of ewes, also, the birth rate has been improved. However, the ewe’s lactation could not meet the needs of the lambs after 3 weeks, severely restricting the development of the lambs. Therefore, supplementary feeding starter is needed as soon as possible to meet the needs of lamb’s rapid growth. Starter contains digestible, comprehensive, rich, and palatable nutrients. The nutrient level of the starter is crucial for the growth and development of rumen of young ruminants. Li et al. (2015b); Li et al. (2015a) found that rumen digestion and development began at the age of 7 days, and milk replacer or the starter can affect the rumen development and its microbial community at 21 days old. What’s more, starter can promote the metabolism of volatile fatty acids (VFA) in ruminal epithelium in pre-weaned lambs (Sun et al., 2018). The concentration of total VFA, acetate, butyrate, propionate, valerate, isobutyrate and isovalerate increased, and the length and width of the rumen papillae increased significantly after supplementary feeding starter (Malmuthuge, Liang & Guan, 2019). Additional nutrients of concentrates feed can manipulate the core community structure to produce more VFA, which ultimately promote rumen development and function in goats (Lv et al., 2019).

In this study, 22 Hu lambs were selected and the effect of starter feeding on rumen of lambs was analyzed by 16S amplification method. In order to make the results more detailed, we systematically analyzed the rumen morphology, internal environment and microorganisms. To a large extent, this study can provide references for the follow-up studies on the starter and early rumen development of lambs. Our aims were as follows: (i) to determine the effects of early supplementary feeding on rumen morphology and internal environment; (ii) to analyze the effects of early supplementary feeding on rumen microbial.

## Materials & Methods

### Ethics statement

The study protocol was reviewed and approved by the Animal Welfare Ethics Committee of Gansu Agricultural University (Approval No. 20180173). And the animal procedures used in this study strictly abide by the Administrative Measures of Gansu Province on Experimental Animals.

### Animal study and sample collection

A total of 22 ((birth weight = 3.65 ± 0.49 kg) (mean ± SD)) healthy neonatal male Hu lambs were selected from a commercial sheep farm (Jinchang Zhongtian Sheep Industry, Co., Ltd., Gansu, China). All lambs were chosen from twin lambs. After the lambs were born, they followed the ewe and eats colostrum for 6 days. When the lambs were 7 days old, they were transferred to the experimental base (Defu Agricultural Science and Technology co., ltd. Gansu, China). During the experiment, the local temperature was 15−26 °C. The Hu lambs were randomly assigned to one of two diets. In the control group (group C), lambs were fed with the milk replacer, while the experimental group (group S) were fed with milk replacer and a starter. The two groups were fed with milk replacer in a certain proportion and time (08.00, 14.00, and 20.00 h every day). At the same time, the group S was weighed and fed with starter at 08.00 h in the morning.

The special milk replacer of lambs (Dry matter: 96.91%, Crude Protein: 23.22%, fat: 13.20%; patent number: ZL02128844.5), which was provided by Feed Research Institute Chinese Academy of Agricultural Sciences (Beijing, China). Before feeding, the milk replacer and water were 1:5 for brewing. Water was available to the Hu lambs ad libitum. The formulation and chemical composition of the starter are shown in Table 1. The starter was specially prepared and made into pellets according to the test requirements and the requirements of “Feeding standard of meat-producing sheep and goats (NYT816-2004)”.

**Table 1:** Ingredient composition and nutritional levels of the starter (air dry basis).

Item (%)		Nutritional level	
Alfalfa hay	30.90	Crude protein (%)	21.13
Corn	11.00	Digestible energy (MJ/kg)	12.02
Extruded corn	15.60	Neutral detergent fiber (%)	25.07
Bran	2.00	Calcium (%)	0.83
Dried malt root	12.00	Phosphorus (%)	0.34
Soybean meal	13.10		
Expended soybean	7.70		
Corn gluten meal	6.00		
Limestone	0.30		
Premix^a^	1.00		
Nacl	0.40		

**Notes.**

a Provided per kilogram of diets: S 200 mg, Fe 20 mg, Zn 25 mg, Cu 2 mg, Mn 8 mg, I 0.24 mg, Se 0.2 mg, Co 0.1 mg, VA 470 IU, VD 50 IU, VE 10 IU, VB1 0.9 mg, VB2 2.4 mg, Pantothenic acid 2.0 mg, Nicotinic20 mg, Biotin 0.2 mg, Folic acid 0.2 mg, VB12 0.01 mg.

When they were the 28 days old, six lambs from each group were randomly selected and sacrificed by neck bleeding before feeding in the morning. The rumen pH was measured using a pH meter (Testo Instruments International Trading (Shanghai) Ltd., Shanghai, China) immediately after slaughter. Rumen abdominal sac tissue were collected and rinsed with normal saline after sacrifice, then fixed in 4% paraformaldehyde for histomorphological analysis (ruminal papillae length and width and the tunica muscularis thickness). Simultaneously, samples of the rumen content were thoroughly mix well and collected into a 5 mL freezer-storage tube with high-temperature sterilization treatment immediately and stored at −80 °C for DNA extraction and further analysis. Since the rumen contents of lamb contained less water, the collected rumen contents were centrifuged to obtain rumen fluid for the detection of rumen fermentation parameters. The rest of the lambs which not slaughtered were sent to the farm. (Defu Agricultural Science and Technology co., ltd. Gansu, China).

### Detection of rumen fermentation parameters and rumen tissue morphology

The concentration of ammonia nitrogen (NH_3_-N) in the rumen fluid was determined using the method described by Feng & Gao (2010). According to the method described by Erwin, Marco & Emery (1961), the concentrations of volatile fatty acids (VFA), acetate, propionate, and butyrate in the rumen fluid were determined using a gas chromatography analyzer (AI 3000, Thermo Fisher Scientific GmbH, Dreieich, Germany). The length, width and muscular thickness of the rumen papilla were measured using a Digital microscope (BA210 Digital, Motic China Group Co., Ltd, China)

### DNA extraction and high-throughput sequencing

Total genomic DNA from the samples was extracted using the cetyltrimethylammonium bromide (CTAB)/sodium dodecylsulfate (SDS) method. DNA concentration and purity were monitored on 1% agarose gels. According to the concentration, DNA was diluted to 1 ng/µL using sterile water. Using the diluted genomic DNA as a template and specific primers with barcodes, New England Biolabs Phusion^®^ High - Fidelity PCR Master Mix with GC Buffer and High Fidelity enzymes for PCR were used to amplify the V4 regions of the 16S rRNA, using the universal primers 515F (5′-GTGCCAGCMGCCGCGGTAA-3′) and 806R (3′-GGACTACHVGGGTWTCTAAT-5′). PCR reactions were carried out in 30 µL reactions with 15 µL of Phusion^®^ High-Fidelity PCR Master Mix (New England Biolabs); 0.2 µM of forward and reverse primers; and approximately 10 ng of template DNA. Thermal cycling consisted of initial denaturation at 98 °C for 1 min; followed by 30 cycles of denaturation at 98 °C for 10 s, annealing at 50 °C for 30 s, and elongation at 72 ° C for 30 s; with a final incubation at 72 °C for 5 min. The PCR products were mixed with same volume of 1 × loading buffer (containing SYB green) and subjected to electrophoresis on 2% agarose gels for detection. PCR products were mixed in equidensity ratios. Then, mixtures of PCR products were purified using a Gene JET™ Gel Extraction Kit (Thermo Scientific). Sequencing libraries were generated using Ion Plus Fragment Library Kit 48 reactions (Thermo Scientific) following manufacturer’s recommendations. The library quality was assessed on a Qubit@ 2.0 Fluorometer (Thermo Scientific). Finally, the library was sequenced on an Ion S5™ XL platform and 400 bp/600 bp single-end reads were generated. Raw data are available through the National Center for Biotechnology Information (NCBI) Sequence Read Archive (SRA) under BioProject accession PRJNA590858.

### Sequence data processing

Single-end reads was assigned to samples based on their unique barcode and truncated by cutting off the barcode and primer sequences and quality filtering of the raw reads was performed under specific filtering conditions to obtain the high-quality clean reads, according to the Cutadapt (Martin, 2011) (V1.9.1, http://cutadapt.readthedocs.io/en/stable/) quality control process. Then, the reads were compared with the reference database (Silva database, https://www.arb-silva.de/) (Quast et al., 2013) using the UCHIME algorithm (UCHIME Algorithm, http://www.drive5.com/usearch/manual/uchime_algo.html) (Edgar et al., 2011) to detect and remove chimeric sequences (Haas et al., 2011), which led to obtain the Clean Reads. Sequences analysis was next performed using the Uparse software (v7.0.1001, http://drive5.com/uparse/) (Edgar, 2013) and ≥ 97% similarity were assigned to the same operational taxonomic units (OTUs).Then the Silva Database (https://www.arb-silva.de/) (Quast et al., 2013) was used, based on the Mothur algorithm, to annotate taxonomic information for each representative sequence.

Multiple sequence alignment was conducted using the MUSCLE software (version 3.8.31, http://www.drive5.com/muscle/) (Edgar, 2004). Alpha diversities (observed-species, Chao1, Shannon, Simpson, ACE, and good-coverage) were calculated using Qiime (version 1.7.0) and displayed using the R software (version 2.15.3). For beta diversity analysis, principal coordinate analysis (PCoA) and unweighted pair group method with arithmetic mean (UPGMA) were performed using Qiime (version 1.7.0) by unweighted unifrac distance. Furthermore, environmental factor correlation analysis was performed using Spearman association analysis. The Spearman correlation index between species and environmental factors was calculated, and the significance was tested using corr.test in the psych package from R, after which the visualization was performed using the pheatmap function in the pheatmap package (Segata et al., 2011).

### Statistical analysis

The phenotypic data (rumen papillae length and width, tunica muscularis thickness, NH3-N content, Acetate, Propionate, Butyrate, TVFA and pH) were subjected to Independent-sample *t* test analysis using SPSS 20.0 (IBM Corp., Armonk, NY, USA). And the data conform to the normal distribution of parameter test, and the homogeneity test of variance is reasonable. Statistical results were presented as means ± SE and the significance was set at *P* < 0.05.

## Results

### Phenotypic variables in Hu lambs of the groups

The phenotypic variables of group C and group S are shown in Table 2 and Table S1. In general, the starter significantly affected the length of the rumen papilla, and the length of the rumen papilla was longer in group S compared with group C (*P* = 0.003). Meanwhile, the concentration of acetate, propionate, butyrate, and TVFA showed significant differences between the two groups, they were all higher than group C (*P*  <  0.001). However, the rumen papillary width and muscular layer thickness were not significantly different between the two groups (*P* >  0.05). The NH_3_-N concentration, the ratio of acetate to propionate, and the pH value also showed no significant differences (*P*  >  0.05).

**Table 2:** Phenotypic variables in Hu lambs of the two groups.

Items	C group	S group	SEM	*P*-value
Rumen papillae				
Length, µm	350.20	539.86	48.19	0.003
Width, µm	246.58	221.94	26.18	0.372
Tunica Muscularis thickness, µm	887.37	1,081.99	217.00	0.406
NH3-N content, mg/100 mL	24.35	27.71	6.56	0.620
Acetate, mmol/L	18.43	61.28	6.69	<0.001
Propionate, mmol/L	6.08	20.77	1.19	<0.001
Butyrate, mmol/L	1.01	13.03	1.63	<0.001
A/P,	3.29	2.94	0.71	0.632
TVFA, mmol/L	29.40	99.20	7.21	<0.001
pH	5.54	5.54	0.21	1.000

### PCR amplification and high-throughput sequencing of bacterial DNA

After sequencing and filtering low-quality and long-section sequences, an average of 85,336 reads were obtained from the 12 samples, and 80,093 valid sequences were obtained on average after quality control. The average length of reads was 253 bp, and the quality control efficiency was 93.92% (Table S2).

### Abundance and diversity analysis of OTUs

#### Analysis and species annotation of OTUs

**Figure 1:**
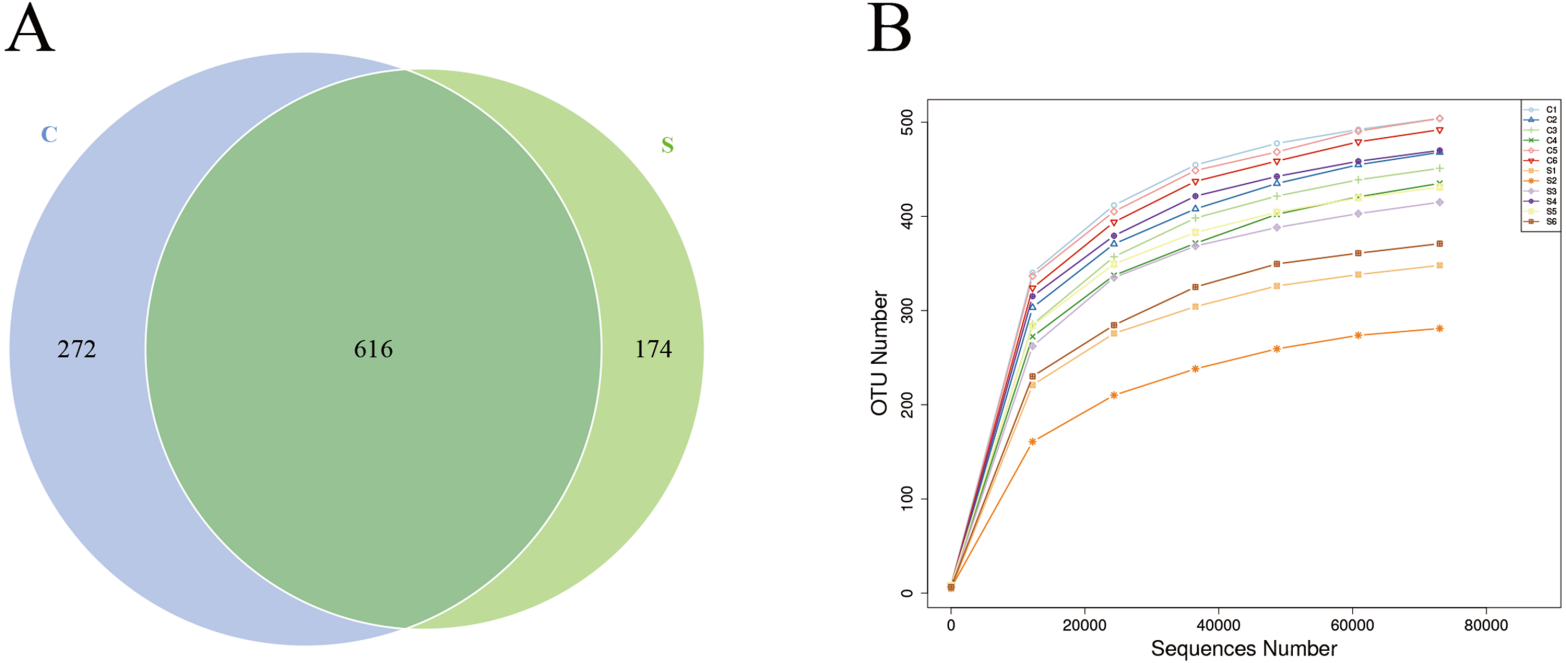
Number of operational taxonomic units (OTUs) in each group. (A) Venn diagram of shared OTUs; (B) rarefaction curves of OTUs.

Sequences were clustered into OTUs with 97% identity, obtaining a total of 1,064 OTUs. Among them, 888 OTUs were obtained for group C and 790 OTUs were obtained for group S, among which 616 OTUs were shared by both groups (Fig. 1A), accounting for 69.37% of the total OTUs in group C and 77.97% of the total OTUs in group S. Next, rarefaction curves were plotted to determine whether the current sequencing depth of each sample was sufficient to reflect the microbial diversity included in the community sample. The rarefaction curves of all samples obtained under the condition of 97% similarity tended to be flat, indicating that the sequencing data volume is reasonable (Fig. 1B).

**Table 3:** Alpha diversity measures of bacterial communitites.

Items	C group	S group	SEM	*P*-value
observed_species	475.67	386.00	29.89	0.013
shannon	5.16	4.14	3.50	0.015
simpson	0.92	0.83	0.04	0.058
chao1	516.78	410.39	31.45	0.012
ACE	520.29	414.46	30.14	0.011

#### Alpha diversity analysis

As shown in Table 3, Observed_species, shannon, chao1 and ACE in group C were significantly higher than that of group S (*P* = 0.013, *P* = 0.015, *P* = 0.012, *P* = 0.011), the simpson was not significant (*P* = 0.058), but on the numerical value is great than group S. So there were significant differences in the microbiota diversity and richness between the two groups, indicating a higher diversity in group C.

#### Bacteria composition analysis

**Figure 2:**
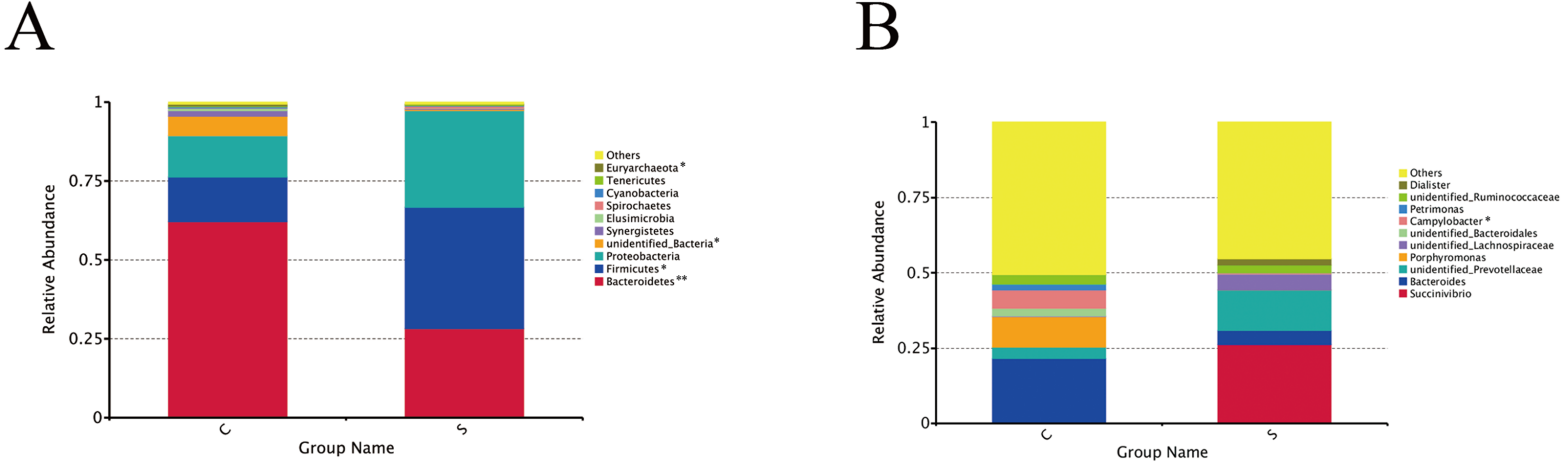
Changes in microorganisms in the rumen of the different groups. Microbial composition at the phylum level (A) and genus level (B). Each bar represents the average relative abundance of each bacterial taxon within a group. ^∗^*P* < 0.05, ^∗∗^*P* < 0.01.

A total of 24 phyla and 230 genera were identified in the rumen microbiome. The most abundant phyla and 10 genera are presented in Fig. 2. At the phyla level (Fig. 2A), the dominant phyla in group C were Bacteroidetes (62.10%), Firmicutes (14.17%), and Proteobacteria (13.13%), accounting for 89.41% of the total phyla. The dominant phyla in group S were the same, but Bacteroidetes (28.12%), Firmicutes (38.48%), and Proteobacteria (30.64%) accounted for 97.33% of the total phyla. Moreover, the Bacteroidetes in group C were present in significantly higher numbers than in group S (*P* = 0.001), while there were significantly more Firmicutes in group S than in group C (*P* = 0.043).

At the genus level (Fig. 2B), the genera annotated in group C were *Bacteroides* (21.43%), *Porphyromonas* (10.13%), and *Campylobacter* (6.14%), followed by *Succinivibrio* (0.11%), *unidentified_Prevotellaceae* (3.80%), *unidentified_Lachnospiraceae* (0.15%), *Petrimonas* (1.86), and *Dialister* (0.02%), which accounted for 49.49% of the total genera in group C. *Succinivibrio* (26.21%), *unidentified_Prevotellaceae* (13.52%) and *unidentified_Lachnospiraceae* (5.30%) were first three dominant bacterial genera in group S, followed by *Bacteroides* (4.57%), *Porphyromonas* (0.07%), *unidentified_Bacteroidales* (0.01%), *Campylobacter* (0.38%), *Petrimonas* (0.01%), *unidentified_Ruminococcaceae* (2.42%), and *Dialister* (2.17%), which accounted for 54.66% of the total genera.

#### Beta diversity analysis

**Figure 3:**
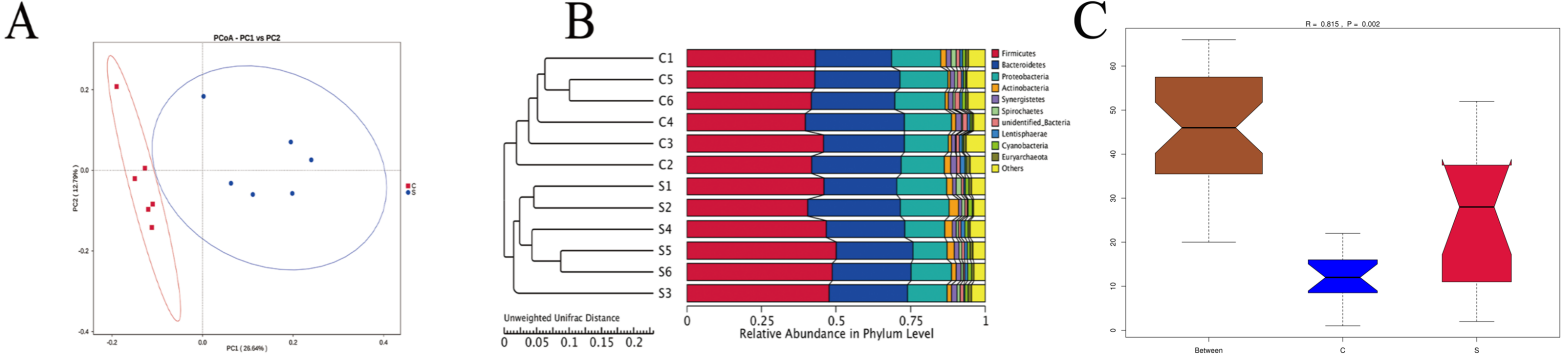
Unweighted Unifrac distance based on thePrincipal coordinate analysis (PCoA) (A), UPGMA (unweighted pair group method with arithmetic mean) clustering graph (B) and Anosim analysis (C). *R*-value is between (−1, 1), and R-value is greater than 0, indicating significant difference between groups, or indicating that the intra-group difference was greater than the inter-group difference, and *P* < 0.05 indicated statistical significance.

The results (Fig. 3A) showed that the six samples from group C were distributed compactly, the samples in group S were also clustered together, and the two groups were distributed independently. Based on unweighted unifrac principal coordinates, the sharing of the first and second principal components was 26.64% and 12.79%, respectively. An unweighted unifrac distance matrix was made using a UPGMA cluster analysis, and the 12 samples of group C and group S were clustered hierarchically to judge the similarity of the species composition among the samples. Figure 3B shows that the six biological replicates of group C and group S were first clustered together, and then species composition and richness of group C and group S were judged to be different. And in the analysis of Anosim (Chapman & Underwood, 1999) (Fig. 3C), we can see that *R* = 0.815, *P* = 0.002, indicating that the difference between group C and group S is significant and statistically significant. Moreover, the grooves of group C and group S do not overlap, indicating that their median is also significantly different.

### LEfSe analysis of intergroup samples

**Figure 4:**
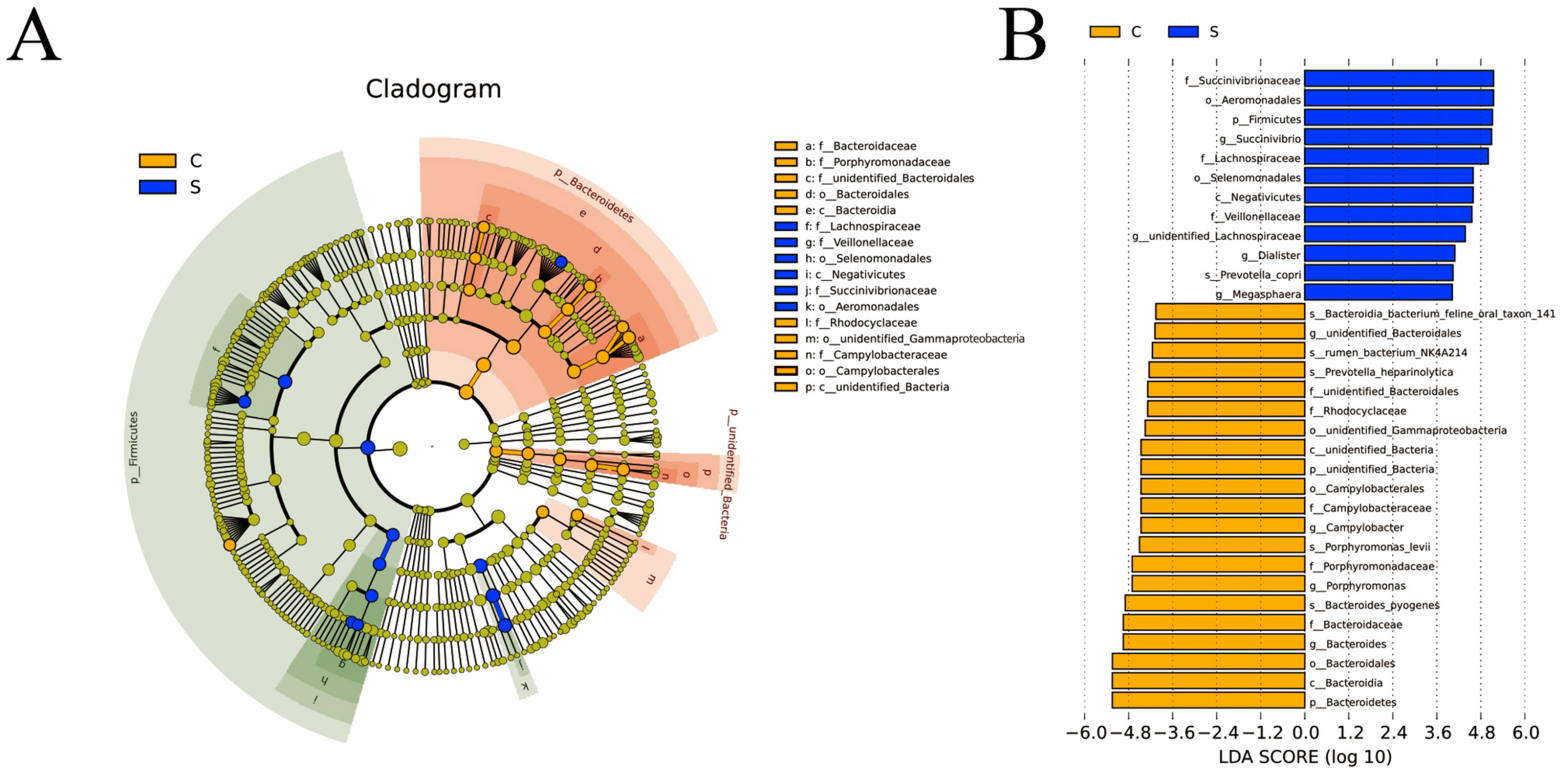
Cladogram (A) and linear discriminant analysis (LDA) (B) value distribution histogram. Bacterial taxa significantly differentiated between the C and S groups identified by LDA coupled with effect size (LEfSe) using the default parameters.

LEfSe software was used for LEfSe analysis on the basis of OTUs level and the filter value of LDA Score is 4. In group C, *Porphyromonadaceae* and *unidentified_Bacteroidales* of the Bacteroidetes, *Campylobacteraceae* of the Campylobacterales were identified as important. In group S, *Lachnospiraceae* and *Veillonellaceae* of the Selenomonadales and *Negativicute* and *Succinivibrionaceae* of the Aeromonadales were identified as important (Fig. 4A). The LDA histogram (Fig. 4B) showed that there were 33 LDA scores with >4 biomarkers, including 21 in group C, including *unidentified_Bacteria*, *Bacteroidetes*, *unidentified_Bacteroidales*, *Campylobacter*, *Porphyromonas*, and *Bacteroides*; and 12 in S group, including *Firmicutes*, *Succinivibrio*, *unidentified_Lachnospiraceae*, *Dialister*, and *Megasphaera*.

### Relationship between the bacterial community and phenotypic variables

**Figure 5:**
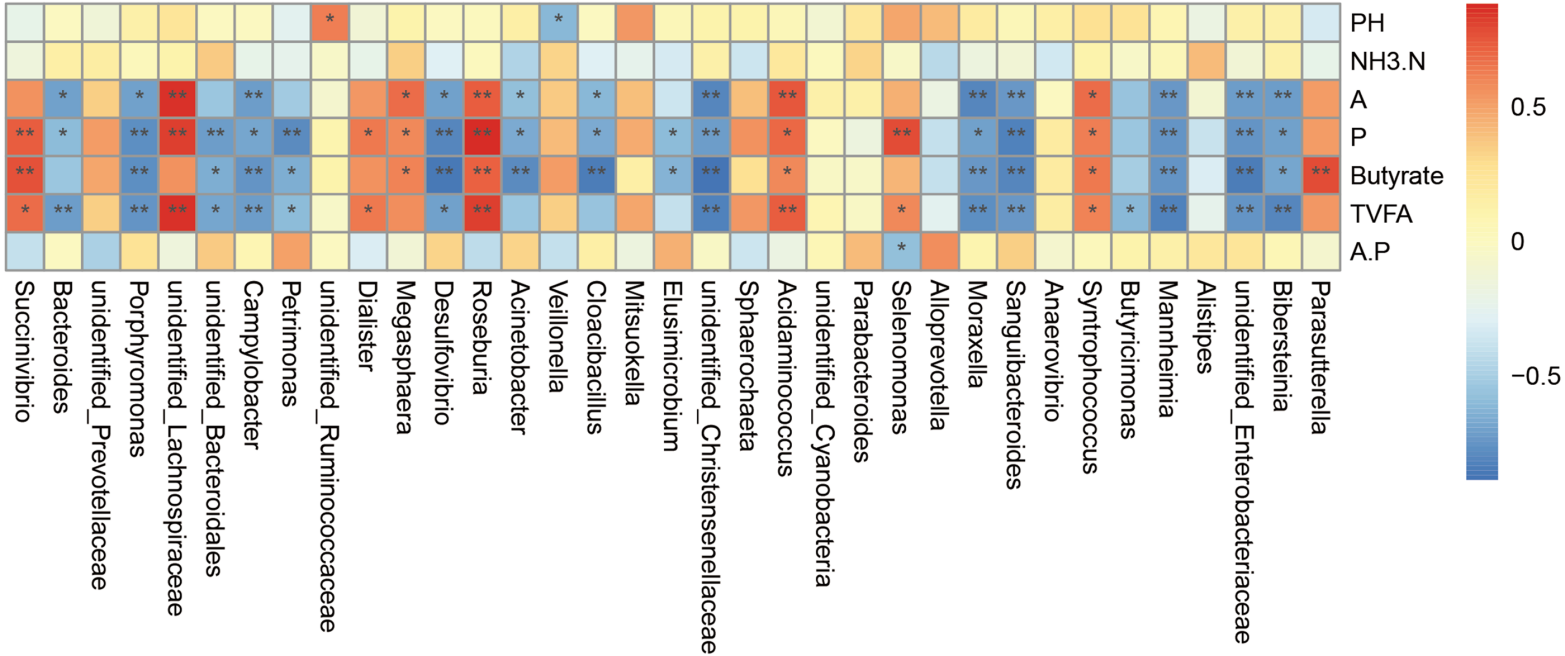
Spearman’.s correlation between the rumen bacterial communities (genus level) and phenotypic variables. Red represents a positive correlation and blue represents a negative correlation. A is meaning Acetate, P is meaning Propionate, A.P is meaning Acetate/ Propionate. ^∗^ *P* < 0.05, ^∗∗^ *P* < 0.01.

The correlation analysis was carried out on the genus abundance. Correlation analysis (Fig. 5) showed that the relative abundances of *Porphyromonas*, *Campylobacter*, *Desulfovibrio, unidentified_Christensenellaceae, Moraxella, Sanguibacteroides, Mannheimia, unidentified_Enterobacteriaceae* and *Bibersteinia* correlated negatively with the concentrations of acetate, propionate, butyrate, and TVFA; *unidentified_Bacteroidales* and *Petrimonas* correlated negatively with the concentrations of propionate, butyrate, and TVFA; *Acinetobacter* and *Cloacibacillus* correlated negatively with the concentrations of acetate, propionate, and butyrate; *Bacteroides* correlated negatively with the concentrations of acetate, propionate, and TVFA (*P* <  0.05). While the relative abundances of *Roseburia, Acidaminococcus*, and *Syntrophococcus* correlated positively with the concentrations of acetate, propionate, butyrate, and TVFA; *Succinivibrio* correlated positively with the concentrations of propionate, butyrate, and TVFA; *unidentified_Lachnospiraceae* correlated positively with the concentrations of acetate, propionate, and TVFA, *Dialister* correlated positively with the concentrations of acetate and TVFA; *Megasphaera* correlated positively with the concentrations of acetate, propionate, and butyrate; and *Selenomonas* correlated positively with the concentrations of acetate and TVFA (*P* < 0.05).

## Discussion

Early development of the rumen affects the digestive capacity and production of adult lambs, and rumen development could be measured by the developmental status (length and width) of the rumen papilla (Lesmeister, Heinrichs & Gabler, 2004). There are many reports on the rumen tissue morphology of ruminants; however, the results vary greatly because of the differences in feed nutrition level or composition (Álvarez-Rodríguez et al., 2012). In the present study, the length of the rumen papillae in the supplementary (starter) feeding group was significantly higher than that in the milk replacer group, while the rumen papillae width was lower than that in the milk replacer group. This is similar to the report of Wang (2013) in which the dietary concentrate level of calves increased, and the rumen papillae width decreased. Colonization by rumen microorganisms and the existence of fermentation substrates are the basis of rumen fermentation activities. Rumen pH, the concentration of NH_3_-N and VFA of ruminants are all important indicators of fermentation, which can reflect rumen function, regulate the acid and base balance, and the electrolyte balance in the internal environment. In this experiment, there was no difference in pH between the two groups. This may be because although the rumen development of lambs in the supplementary (starter) feeding group was better than that in the milk replacer group, the ability of the rumen wall of lambs to absorb and transport VFA was limited, and the frequency of rumination and the dilution of pH by saliva were also limited. Studies have shown that VFA can be detected in the rumen of calves at 2 weeks of age (Beharka et al., 1998), and the VFA concentration can reach the level of adult animals at 8 weeks of age. There were significant differences in the concentrations of acetate, propionate, butyrate, and TVFA between the two groups, all of which were significantly greater in supplementary feeding group than in the milk replacer group. Some studies have shown that increased intake of starter by lambs would increase the carbohydrates fermented by microorganisms in the rumen, thus leading to increased concentrations of VFA (Bach et al., 2007; Vi Baldwin et al., 2004). In addition, after supplemental feeding, the concentrated feed would be decomposed by rumen microorganisms, producing a large amount of propionate and butyrate, and the corresponding proportion of acetate would decrease, which was in line with rumen microbial fermentation law. Moreover, microbial changes at the phylum and genus levels were consistent with this. NH_3_-N is the rumen’s endogenous nitrogen, and is a feed and non-protein nitrogen decomposition end product. Currently, it is generally believed that the appropriate NH_3_-N concentration range for rumen microbial growth is 5–28 mg/dL (Wanapat & Pimpa, 1999). In the present study, although the difference in the NH_3_-N concentration between the two groups was not significant, the NH_3_-N concentration was in the suitable range for fermentation, which indicated that the rumen microorganisms had a strong ability to decompose protein in the feed, and the microbiota had been established. In addition, the results of this study were similar to those of lambs slaughtered at 42 days after supplementary feeding (Wang et al., 2016), indicating that early supplementary feeding promotes rumen development in lambs.

Based on the 16S rRNA V4 highly variable region, second-generation high-throughput sequencing technology was used to analyze the diversity of microorganisms, which not only reduces the separation and cloning errors of microorganisms, but also provides relatively complete information for the microbiota. Therefore, it is widely used to study environmental microbial diversity. Rumen microorganisms are mainly composed of fungi, bacteria, and protozoa, among which the species and quantity of bacteria vary, but account for the vast majority of the total microorganisms. Rumen bacteria can digest carbohydrates in the rumen and produce nutrients for the host, such as VFA, microbial proteins, and vitamins. The diet is the main factor affecting the structure and function of the rumen microbial community (Kocherginskaya, Aminov & White, 2001; Yáñez Ruiz, Abecia & Newbold, 2015). Using the Ion S5™ XL sequencing platform, we obtained an average of 85,336 reads (>93%) of each well-covered sample. The number of OTUs in the milk replacer group was significantly greater than that in the supplementary feeding group, and the analysis results of Alpha and Beta diversity also showed that there was a significant difference in the rumen microbial community between the two groups after supplementary feeding. The rumen microbial diversity decreased after supplementary feeding, which was consistent with the results of previous research (Wang et al., 2016). Therefore, the structure of the rumen microbiota will change if a lamb is given early supplementary feeding. The diversity of the rumen microbiota was conducive to the stability of the rumen environment; however, the diversity of rumen microbiota in the supplementary feeding group was lower, this is probably because with the intake of starter, the dominant bacteria were established, however some were disappeared, which is similar to the results of our previous research group (Wang et al., 2016). It is indicating that the establishment of rumen microbiota in early ruminants is a more complex process. In recent years, the colonization of rumen microorganisms in young ruminants has attracted increased attention from scholars at home and abroad (Li et al., 2012; Jami et al., 2013). Yang (2017) found that at week 0, the microbial diversity of rumen contents was established in lambs only fed with milk replacer. Studies have shown that in vertebrates, especially in mammals, the dominant bacteria are Proteobacteria, Firmicutes, and Bacteroidetes (Ley et al., 2008). In the present study, the dominant bacteria of the two groups were the same, which was consistent with the previous results (Ley et al., 2008; Qin et al., 2010; Malmuthuge et al., 2013). However, the abundance of Firmicutes in the supplementary feeding group (38.48%) was significantly higher than that in the milk replacer group (14.17%) (*P* = 0.043), while the abundance of Bacteroidetes (28.21%) was significantly lower than that in the milk replacer group (62.10%) (*P* = 0.001). The relative abundance of *Succinivibrio* and *unidentified_Prevotellaceae* in the supplementary feeding group was 39.74%, while it was only 3.91% in the milk replacer group. This phenomenon might reflect the higher fiber content in the supplementary feed starter compared with that in the control feed, and the Firmicutes are the main bacteria that decompose fibers, including a large number of bacteria which can decompose cellulose (Matsui et al., 2000), such as *Buiyrivibrio, Ruminococcus, Pseudobutyrivibrio, Oscillibacter* and *Eubacterium*. These bacteria can promote the decomposition of cellulose (Brulc et al., 2009) and the fermentation of polysaccharides, thus leading to an increase in their relative abundance. Bacteroidetes are closely related to the conversion of DNA, proteins, lipids, and other organic substances, and participate in the metabolism of saccharide, bile acids and steroids (Salyers, 1984; Michaud et al., 2009). The increase of Bacteroidetes in the milk replacer group also occurred when human babies were fed formula (Thompson, 2012). *Prevotella* bacteria efficiently hydrolyze starch and protein (Wu et al., 2012). It has been reported that *Prevotella* can account for 60–70% of rumen microorganisms (Meyer, Stenzel & Hofreiter, 2008). In addition, *Prevotella* has the ability to digest and use starch, xylan and pectin (Evans et al., 2011; Kopecný et al., 2003). *Succinivibrio* can also ferment a variety of sugars to produce acetate and propionate. Thus, the increase in the concentrations of acetate, propionate, butyrate, and total acids in the supplementary feeding group were understandable, because as the abundance of microorganisms associated with carbohydrate fermentation increased, so did the production of volatile fatty acids.

We performed correlation analysis at the genus level and found that both positive and a negative correlations. For example, *Bacteroidetes* were negatively correlated with the concentration of acetate, propionate, and TVFA. *Bacteroidetes*, as the main member of intestinal microbial community, can degrade complex plant or animal sugars and produce a large number of short-chain fatty acids, which provide nutrients and calories to benefit the host (Wexler, 2007; Koropatkin, Cameron & Martens, 2012). This might be caused by the increased abundance of *Prevotella* of the Bacteroidetes after supplementary feeding. The abundance of Bacteroidetes in the milk replacer group was 21.43% and the abundance of *Prevotella* was 3.80%, compared with 4.57% and 13.52% in the supplementary feeding group. *Succinivibrio* was positively correlated with the concentration of propionate, butyrate, and TVFA. *Dialister* was positively correlated with the concentration of acetate and TVFA. Although *Succinivibrio* does not ferment carbohydrates and amino acids, it has the ability to degrade the activity of fructan and protein in herbage, and the fermentation products mainly include acetate and succinate, which can be converted into propionate to promote the formation of bacterial proteins. A few strains can also secrete active enzymes that can decompose plant cell walls (Van Gylswyk, 1995; Li et al., 2015b; Li et al., 2015a). Studies have also shown that *Succinivibrio* and *Roseburia* levels seem to increase through a grain-rich diet and might contribute to the fermentation of a variety of unstructured carbohydrates (Henderson et al., 2015; Plaizier et al., 2017). *Roseburia* is also known as a producer of butyrate (Jiang et al., 2016). In the present study, after supplementary feeding, both *Succinivibrio* and *Roseburia* levels were increased, which was consistent with the change trend of the concentration of acetate, propionate, and butyrate. *Megasphaera* was positively correlated with the concentration of acetate, propionate, and butyrate. *Megasphaera elsdenii* is the major lactate decomposition bacteria in the rumen. When the rumen pH value decreases to 5.5, the activity of most lactate decomposing bacteria is inhibited; however, they could also utilize lactate for metabolism, and more than 70% of lactate in the rumen could be decomposing and utilized, and the products were mainly propionate, butyrate, and a small amount of acetate and valeric acid (Asanuma & Hino, 2002), which was also consistent with the observed change of VFA. VFA can enhance the papilla of the rumen and promote its development. After supplementary feeding in this study, the increase in VFA levels significantly promoted the development of the rumen papilla. *Selenomonas* is also a beneficial genus, and is the main lactate decomposing bacteria in ruminants. It can produce propionate from lactate and maintains a stable pH value. *Campylobacter* and *Desulfovibrio* correlated negatively with various volatile fatty acids. *Campylobacter* is associated with various veterinary diseases (Behling, Pham & Nowotny, 1979; Humphrey & Beckett, 1987). Stanley et al. (1998a); Stanley et al. (1998b) data supported the growth of *Campylobacter* in the rumen, especially in young animals (Stanley & Jones, 2003). *Desulfovibrio* is a sulfate reducing bacteria and relatively common pathogenic bacteria in the intestinal tract. It can reduce sulfate to sulfide in the intestinal tract, which has a toxic effect on intestinal epithelial cells and can induce their abnormal proliferation and metabolism (Gill et al., 2006). Inhibition of butyrate oxidation leads to destruction of the intestinal barrier function (Eckburg et al., 2005). After supplementary feeding, the abundance of both genera decreased significantly, which also indicated that the incidence of disease of lambs could be reduced after supplementary feeding. The links between microorganisms are numerous; therefore, it is difficult to achieve clarity, because most of the microorganisms in the rumen have symbiotic and antagonistic effects, some of which produce metabolites that are nutrients for other microorganisms, while others are part of an extended food chain.

## Conclusions

In summary, early supplementary feeding of the lambs can not only promote the development of the rumen, but also promote the production of certain short-chain fatty acids, such that the environment in the rumen changes, which causes a series of changes in the microorganisms in the rumen. In the present study, the rumen microbial diversity in the milk replacer group was greater than that of the starter feeding group. *Bacteroides, Succinivibrio,* and *Roseburia* genera were closely associated with the concentration of TVFA, acetate, propionate and butyrate, showing positive or negative correlations. However, the causal relationship between the host and the microbial communities are unclear, and remains to be further explored. There were different microbes in each group. The number of microorganisms is huge, and their connections and relationships are complex and changeable. Therefore, more effort should be made to further understand the microbial community. Our results suggest that, in the early feeding of lambs, a starter should be supplemented as early as possible to promote the development of rumen tissue morphology and the microorganisms in the rumen.

##  Supplemental Information

10.7717/peerj.9353/supp-1 Supplemental Information 1 Phenotypic variables in Hu lambs of the two groups

10.7717/peerj.9353/supp-2 Supplemental Information 2 Evaluation results of the sequencing data for each sample Raw Reads refers to the sequences filtered for low quality and short length; Clean Reads refers to the sequences filtered for chimeras, which were used for subsequent analysis; Base refers to the number of bases in the final Clean Reads; AvgLen refers to the average length of the Clean Reads; the percentage of bases with base mass value greater than 20 (sequencing error rate less than 1%) in Q20 Clean Reads; GC (%) represents the content of GC bases in Clean Reads. Effective (%) represents the percentage of the number of Clean Reads and the number of Raw Reads.

10.7717/peerj.9353/supp-3 Supplemental Information 3 The original sequencing data of S1 in group S used for sequence data processing and analysis

10.7717/peerj.9353/supp-4 Supplemental Information 4 The original sequencing data of S2 in group S used for sequence data processing and analysis

10.7717/peerj.9353/supp-5 Supplemental Information 5 The original sequencing data of S3 in group S used for sequence data processing and analysis

10.7717/peerj.9353/supp-6 Supplemental Information 6 The original sequencing data of S4 in group S used for sequence data processing and analysis

10.7717/peerj.9353/supp-7 Supplemental Information 7 The original sequencing data of S5 in group S used for sequence data processing and analysis

10.7717/peerj.9353/supp-8 Supplemental Information 8 The original sequencing data of S6 in group S used for sequence data processing and analysis

10.7717/peerj.9353/supp-9 Supplemental Information 9 The original sequencing data of C1 in group C used for sequence data processing and analysis

10.7717/peerj.9353/supp-10 Supplemental Information 10 The original sequencing data of C2 in group C used for sequence data processing and analysis

10.7717/peerj.9353/supp-11 Supplemental Information 11 The original sequencing data of C3 in group C used for sequence data processing and analysis

10.7717/peerj.9353/supp-12 Supplemental Information 12 The original sequencing data of C4 in group C used for sequence data processing and analysis

10.7717/peerj.9353/supp-13 Supplemental Information 13 The original sequencing data of C5 in group C used for sequence data processing and analysis

10.7717/peerj.9353/supp-14 Supplemental Information 14 The original sequencing data of C6 in group C used for sequence data processing and analysis

## Additional Information and Declarations

### Data Availability

The following information was supplied regarding data availability: The data is available at NCBI: BioSample, SAMN13341552, BioProject, PRJNA590858.
