# Supplementary material for: Effects of supplementary feeding on the rumen morphology and bacterial diversity in lambs
Source: PeerJ. 2020 Jun 19;8:e9353. doi: 10.7717/peerj.9353 (PMC7307561; doi:10.7717/peerj.9353)
Supplement: Supplemental Information 2 — Raw Reads refers to the sequences filtered for low quality and short length; Clean Reads refers to the sequences filtered for chimeras, which were used for subsequent analysis; Base refers to the number of bases in the final Clean Reads; AvgLen refers to the average length of the Clean Reads; the percentage of bases with base mass value greater than 20 (sequencing error rate less than 1%) in Q20 Clean Reads; GC (%) represents the content of GC bases in Clean Reads. Effective (%) represents the percentage of the number of Clean Reads and the number of Raw Reads. [file peerj-08-9353-s002.docx]

Supplemental Table S2:

Evaluation results of the sequencing data for each sample

| Sample_name | Raw_reads | Clean_Reads | Base (nt) | AvgLen (nt) | Q20 | GC% | Effective% |
| --- | --- | --- | --- | --- | --- | --- | --- |
| C1 | 86,848.00 | 80,074.00 | 20,321,088.00 | 253.00 | 89.49 | 52.95 | 92.20 |
| C2 | 82,569.00 | 80,117.00 | 20,318,329.00 | 253.00 | 89.40 | 54.32 | 97.03 |
| C3 | 83,922.00 | 80,047.00 | 20,292,681.00 | 253.00 | 90.70 | 51.16 | 95.38 |
| C4 | 87,962.00 | 80,062.00 | 20,279,781.00 | 253.00 | 88.16 | 53.35 | 91.02 |
| C5 | 84,376.00 | 80,223.00 | 20,346,943.00 | 253.00 | 90.54 | 50.54 | 95.08 |
| C6 | 85,779.00 | 80,131.00 | 20,327,556.00 | 253.00 | 90.16 | 50.60 | 93.42 |
| S1 | 82,839.00 | 80,174.00 | 20,313,576.00 | 253.00 | 90.69 | 53.21 | 96.78 |
| S2 | 87,255.00 | 80,019.00 | 20,281,252.00 | 253.00 | 91.13 | 51.91 | 91.71 |
| S3 | 87,323.00 | 80,134.00 | 20,325,660.00 | 253.00 | 90.71 | 52.73 | 91.77 |
| S4 | 83,610.00 | 80,038.00 | 20,292,956.00 | 253.00 | 90.83 | 52.88 | 95.73 |
| S5 | 88,778.00 | 80,076.00 | 20,293,171.00 | 253.00 | 89.50 | 55.15 | 90.20 |
| S6 | 82,774.00 | 80,026.00 | 20,270,239.00 | 253.00 | 89.70 | 55.17 | 96.68 |

Raw Reads refers to the sequences filtered for low quality and short length; Clean Reads refers to the sequences filtered for chimeras, which were used for subsequent analysis; Base refers to the number of bases in the final Clean Reads; AvgLen refers to the average length of the Clean Reads; the percentage of bases with base mass value greater than 20 (sequencing error rate less than 1%) in Q20 Clean Reads; GC (%) represents the content of GC bases in Clean Reads. Effective (%) represents the percentage of the number of Clean Reads and the number of Raw Reads.
